# The Role of Ultrasound Features in Predicting the Breast Cancer Response to Neoadjuvant Chemotherapy

**DOI:** 10.7759/cureus.49084

**Published:** 2023-11-20

**Authors:** Mohamed T El-Diasty, Ghofran A Ageely, Sara Sawan, Razan M Karsou, Salwa I Bakhsh, Ahmed Alharthy, Yasser Noorelahi, Arwa Badeeb

**Affiliations:** 1 Radiology, King Abdulaziz University Hospital, Jeddah, SAU; 2 Radiology, Medicine, Rabigh Medical College, King Abdulaziz University, Jeddah, SAU; 3 Radiology, Dalhousie University, Hallifax, CAN; 4 Radiology, King Abdulaziz Hospital, Jeddah, SAU; 5 Pathology, King Abdulaziz University Hospital, Jeddah, SAU; 6 Radiology, King Abdulaziz University, Jeddah, SAU; 7 Radiology, Faculty of Medicine, King Abdulaziz University, Jeddah, SAU

**Keywords:** breast cancers, breast cancer research, breast cancer imag, breast cancer management, ultra sound, neo-adjuvant chemotherapy

## Abstract

Background

Neoadjuvant chemotherapy (NACT) has become the standard of care for locally advanced breast cancer. This study investigates whether baseline ultrasound features can predict complete pathological response (pCR) after NACT.

Methods

This retrospective study was approved by the Institutional Review Board of King Abdulaziz University Hospital, Jeddah, Saudi Arabia, with a waiver of informed consent. Records of female patients aged over 18 years with locally advanced breast cancer treated with NACT from 2018 to 2020 were reviewed. Baseline ultrasound parameters were assessed, including posterior effect, echo pattern, margin, and maximum lesion diameter. Tumor grade and immunophenotype were documented from the core biopsy. pCR was defined as the absence of invasive residual disease in the breast and axilla. Univariate and multivariate analyses assessed the association between ultrasound features and pathological response.

Results

A total of 110 breast cancer cases were analyzed: 36 (32.7%) were estrogen receptor (ER)-positive/human epidermal growth factor 2 (HER-2) negative, 49 (44.5%) were HER-2 positive, and 25 (22.7%) were triple-negative (TN). A pCR was achieved in 20 (18%) of cancers. Lesion diameter was significantly different between pCR and non-pCR groups, 28.5 ± 12 mm versus 39 ± 18 mm, respectively, with an area under the curve (AUC) of 0.7, a confidence interval (CI) of 0.55-0.81, and a p-value of 0.01. No significant association was observed between ultrasound features, tumor grade, and immunophenotype with pCR.

Conclusion

Ultrasound features could not predict pCR. A smaller tumor diameter was the only significant factor associated with pCR. Further prospective studies combining imaging features from different modalities are needed to explore the potential of varying imaging features in predicting post-NACT pathological response more comprehensively.

## Introduction

The introduction of neoadjuvant chemotherapy (NACT) in the treatment of locally advanced breast carcinoma aims to downstage tumors, minimize the recurrence of local disease, and reduce the extent of surgical resection required. Achieving a complete pathological response (pCR) with NACT might also negate the need for surgical interventions such as lumpectomy or mastectomy in some patients [1].

Numerous studies have explored factors predictive of pCR, examining elements like pathological subtypes, immunophenotype, and tumor grade from core biopsies [1-3]. Others have investigated the morphological attributes of original tumors through mammography, ultrasound (US), and magnetic resonance imaging (MRI), and some have compared complete clinical response (cCR) with pCR. Such morphological, histological, and molecular predictors allow a tailored therapeutic approach to administering NACT instead of proceeding directly to surgery [4,5].

Studies have drawn correlations between baseline imaging features and pCR. MRI scans of triple-negative (TN) breast cancers have shown that well-defined and round/oval tumors and the absence of peri-tumoral edema are associated with an improved response to NACT [2]. Another study found that microcalcification in initial mammograms of TN breast cancers was significantly linked with residual disease [3]. A study demonstrated that round tumor shape, posterior acoustic enhancement, and absence of calcifications on baseline US were especially observed among tumors showing pCR [4]. Moreover, a recent study reported that pCR was more likely to occur without mammographic spiculations and posterior acoustic shadowing [5].

At present, only tumor grading and immunophenotypes are routinely considered predictors. Yet, even with these, there is variability in tumor responses within the same immunophenotype or grade. The patient's genomic profile is a crucial factor to consider in tumor behavior.

Since US is routinely performed at baseline for all locally aggressive breast cancers, identifying baseline US features associated with pCR would be invaluable. Therefore, our study aims to investigate whether baseline US features can predict pCR after NACT.

## Materials and methods

Patient selection

The Institutional Review Board of King Abdulaziz University Hospital approved this retrospective study, and the requirement of written informed consent was waived. We searched the hospital's electronic records for patients with locally advanced breast cancer who received NACT between January 2018 and January 2020.

Inclusion criteria included the availability of baseline breast US examination, the results of US-guided core biopsy, the completion of the NACT course, and the completion of mastectomy or lumpectomy with available post-operative histopathologic results. Patients with metastatic disease at the time of diagnosis, known other primary tumors, and those without final histopathological results were excluded.

Ultrasound examinations and analysis

The US examinations were performed by two trained breast sonographers (with 18 and 16 years of experience) utilizing a Philips EPIQ 7 US unit with a high frequency (10-15 MHz) linear transducer. Static longitudinal and transverse images were obtained through all masses. All images were stored in our picture archiving and computer system (PACS).

The ultrasound examinations were retrospectively analyzed by a fellowship-trained breast radiologist (G.A.) with four years of experience in breast imaging. G.A. was blinded to histopathological data. The analyzed US features included maximum tumor size, shape, orientation, echogenicity, margins, predominant acoustic features posterior to the tumors, presence of calcifications, associated distortion, and abnormal axillary LNs.

The findings were assessed based on the ACR BI-RADS US fifth edition [6]. The shape was divided into rounded, oval, and irregular. Orientation was categorized into parallel versus non-parallel. Echogenicity was categorized into anechoic, isoechoic, hyperechoic, hypoechoic, heterogeneous, and complex cystic/solid. Margins were divided into noncircumscribed and circumscribed. Noncircumscribed was further divided into indistinct, angular, microlobulated, and spiculated. The posterior features were divided into four categories: posterior shadowing, posterior enhancement, mixed pattern, and no posterior features.

Tumor classification

Pathological records were retrospectively reviewed by an experienced pathologist (SB with ten years of experience). Histological tumor types, estrogen receptor (ER) and progesterone receptor (PR) status (positive or negative, with positive defined as ≥1%), and human epidermal growth factor 2 (HER2) status (positive or negative) were recorded.

Complete pathologic response was defined as the absence of residual invasive disease in the breast and axilla. The presence of residual invasive disease, including microscopic residual invasive disease, was categorized as a noncomplete response. Residual ductal carcinoma in situ (DCIS) was classified in the complete response group [5].

Statistical analysis

Data were expressed as mean ± standard deviation (SD) for continuous variables and percentages and frequencies for categorical variables. Univariate and multivariable analyses assessed the association between US features and pCR. The chi-square test was used to compare categorical data, while independent samples T-test and receiver operating characteristic (ROC) curve analysis were used to compare continuous data. IBM SPSS Statistics for Windows, Version 24 (Released 2016; IBM Corp., Armonk, New York). was used for the analysis.

## Results

Patient characteristics

A total of 110 patients met the inclusion criteria. The mean age for the study group was 52.7 ± 10 years. A total of 36 (32.7%) of the cases were ER-positive/HER-2 negative, 49 (44.5%) were HER-2 positive, and 25 (22.7%) were triple-negative (TN). A pCR was achieved in 20 (18%) of cancers, while 90 (82%) showed a non-complete response. Baseline characteristics are shown in Table 1.

**Table 1:** Baseline breast carcinoma characteristics in the selected study population (n=110). *Age and tumor size are in mean with SD and range in parentheses. **The rest of the values are in numbers and percentages within parentheses. ER: estrogen receptor, PR: progesterone receptor, HER2: human epidermal growth factor 2.

Characteristic	Value**
Age (years)*	52.7 ± 10 (32-80)
Tumor size (mm)*	37 ± 12 mm (3-100)
Tumor site	
Right breast	62 (56.4%)
Left breast	48 (43.6%)
Tumor grade	
Grade 1	19 (17.3%)
Grade 2	51 (46.3%)
Grade 3	40 (36.4%)
Hormone receptor	
Positive	67 (40%)
Negative	43 (60%)
HER2 receptor	
Positive	49 (44.5%)
Negative	61 (55.5%)
Tumor immunophenotype	
ER/PR positive, HER2 negative	36 (32.7%)
ER/PR negative, HER2 positive	49 (44.5%)
Triple-negative	25 (22.7%)
Pathological response	
Non-complete	90 (81.8%)
Complete	20 (18.2%)

Correlation of US features with tumor grade and hormone receptor status

A summary of baseline US features is provided in Table 2. Due to the small number of patients within some US features, the morphological features were grouped into two main features to achieve greater statistical power. The shape was regular vs. irregular, echogenicity was grouped into hypoechoic versus non-hypoechoic, margins were analyzed as spiculated versus non-spiculated, and posterior features were grouped as shadowing versus non-shadowing.

**Table 2:** The ultrasound (US) characteristics of the breast carcinoma study population (n=110). *Values are in numbers with percentages within parentheses.

US feature	Value*
Orientation	
Parallel	8 (7.3%)
Not parallel	102 (92.7%)
Shape	
Oval	9 (8.2%)
Rounded	5 (4.5%)
Irregular	96 (87.3%)
Margin	
Circumscribed	3 (2.7%)
Indistinct	7 (6.4%)
Angular	29(26.4%)
Lobulated	11(10%)
Spiculated	60(54.5%)
Echogenicity	
Hypoechoic	76 (69.1%)
Heterogeneous	27 (24.5%)
Complex	7 (6.4%)
Posterior features	
No features	38 (34.5%)
Shadowing	45 (40.1%)
Enhancement	8 (7.3%)
Mixed	19 (17.3%)
Calcifications	
Absent	63 (57.3%)
Present	47 (42.7%)
Distortion	
No	11 (10%)
Yes	99 (90%)

Spiculated margins were significantly associated with positive hormone receptors but not tumor grade. Absent calcifications in the US were significantly associated with lower tumor grades (grades 1 and 2 versus grade 3). The remaining features including echogenicity, posterior features, and distortion showed no significant association with hormone receptor status or tumor grade. The Association of US features with hormone receptor status and tumor grade is shown in Table 3. 

**Table 3:** Associations of ultrasound (US) features with hormone receptor status and tumor grade.

Variable	Hormone receptor		P value	Tumor grade		P value
	Negative n=43	Positive n=67		Low (1 and 2) n=70	High (3) n=40	
Margins			0.01			0.55
Spiculations	17	43		40	20	
No spiculations	26	24		30	20	
Echogenicity			1			0.7
Hypoechoic	30	46		47	29	
Not hypoechoic	13	21		23	11	
Posterior features			0.17			0.22
Shadowing	14	31		32	13	
No shadowing	29	36		38	27	
Calcifications			0.32			0.002
Absent	22	41		48	15	
Present	21	26		22	25	
Distortion			0.1			0.5
Absent	7	4		6	5	
Present	36	63		64	35	

Prediction of pathological response

**Figure 1:**
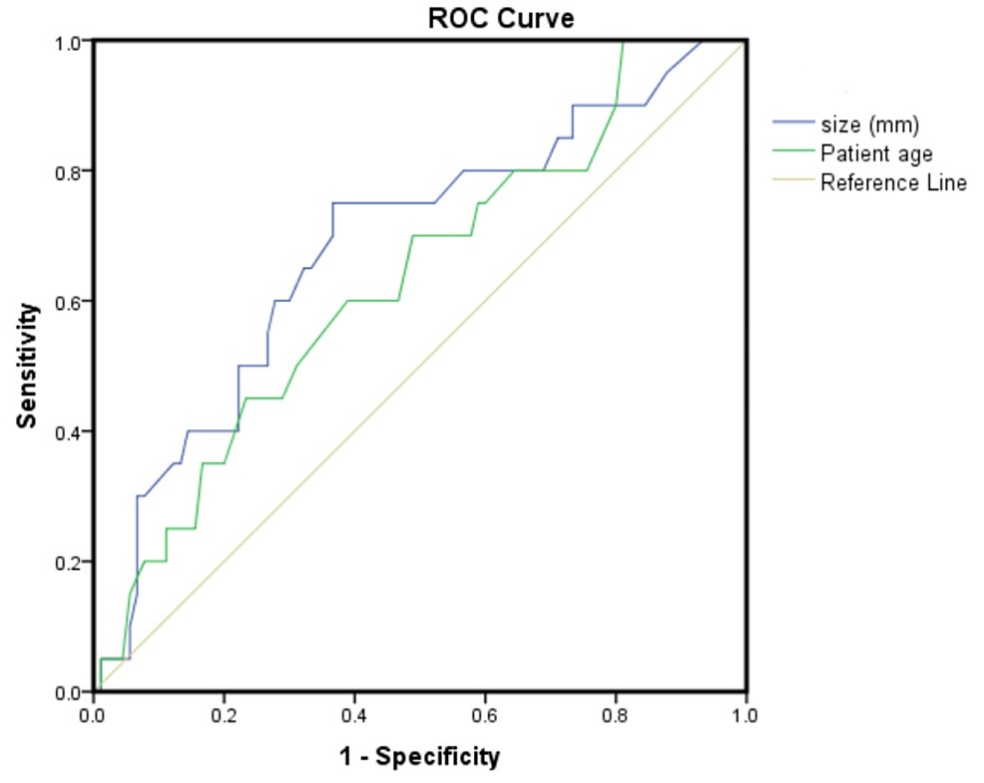
ROC curve of the patient age and tumor size in prediction of pCR. pCR: complete pathological response, ROC: receiver operating characteristic.

The mean patient age in the pCR group was 48.8 ± 9 years compared to 53.7 ± 10 years in the non-pCR group, p = 0.06, AUC = 0.63 (95% CI: 0.49-0.76). Smaller tumor size was significantly associated with pCR (28 ± 14 vs. 39 ± 18 mm) p = 0.01, AUC = 0.69 (95% CI: 0.55-0.81) (Figure 1).

Tumor grade and hormone receptor status were not associated with pathological response. All US features did not show a statistically significant association with pCR. A summary of clinical and US features distribution in pCR and non-pCR groups is shown in Table 4. Though the patient's age reached statistical significance as a predictor of pCR, after multivariate analysis, tumor size was the only significant factor associated with pathological response.

**Table 4:** Associations of pathological response with patients and tumoral demographics.

Variable	Pathological response		P value
	Non-complete (90)	Complete (20)	
Age (years)	53.5 ± 10	48.8 ± 9	0.06
Size (mm)	39 ± 18	28 ± 14	0.01
Tumor grade			0.5
Grade 1	15	4	
Grade 2	44	7	
Grade 3	31	9	
Hormone receptor			1
Positive	55	12	
Negative	35	8	
Margins			0.2
Spiculations	52	8	
No spiculations	38	12	
Echogenicity			1
Hypoechoic	62	14	
Not hypoechoic	28	6	
Posterior features			0.17
Shadowing	14	31	
No shadowing	29	36	
Calcifications			0.08
Absent	48	15	
Present	42	5	
Distortion			1
Absent	9	2	
Present	81	18	

## Discussion

Our study evaluated US imaging characteristics of breast cancer as predictive markers for pCR post-NACT. To our knowledge, such an exploration is not found in the scientific literature of Saudi Arabia. Recognizing the potential impact of geographical demographics and genomic profiles on treatment outcomes, we scrutinized our patient cohort for ultrasound-based predictors of pCR. Our findings indicated that no other US features held any statistically significant correlation with pCR except for tumor size. Specifically, we found a compelling association between smaller tumor size and the likelihood of pCR. Notably, despite identifying a significant link with patient age, only tumor size emerged as a significant factor in our multivariate analysis.

For locally advanced breast cancer, NACT seeks to shrink the tumor to preserve the breast. When NACT lessens the size of the breast tumor or downstages the axilla, it allows for a more cautious operation and enables in vivo assessment of treatment efficacy. NACT modifies the therapeutic or surgical approach depending on patients’ pathological outcomes. Moreover, with NACT, early primary tumor treatment and micro-metastatic illness can be provided [7].

pCR, frequently used as a benchmark endpoint to gauge the effectiveness of NACT, is regarded as a potent predictor of long-term clinical outcomes, such as disease-free status and overall survival [8-10]. Thus, breast cancer management needs to discover and validate variables that predict or enhance pCR rates. In this study, we aimed to evaluate the role of ultrasound features in predicting breast cancer response to NACT.

Our results concur with an earlier study that reported ultrasound features might not be able to predict pCR with enough certainty to suggest therapeutic actions [11]. Another study also concluded that no common imaging methods, ultrasound, mammography, or MRI, are accurate enough to diagnose pCR [12]. Even though there is no agreement on the required negative predictive value (NPV) and false negative rate (FNR), it is clear that the values found in the literature, which range from 51 to 63 and 15 to 39, are not good enough [11,12].

One of the reasons our unadjusted and logistic regression analyses didn’t reveal good predictors of pCR is the small percentage of patients in our study with pCR. Our pCR of 18% is less than the numbers in the literature. Sasanpour et al. reported a 32.9% pCR in an Iranian study [13], while Asaoka et al. [14] found a 24.6% pCR. This is similar to Savaridas et al. [5]; however, they had a larger patient cohort.

Our cohort showed that age and tumor size were statistically different in the complete response and non-complete response groups. Like us, Chou et al. determined that a younger age could predict a pCR [15]. Unlike our study, Baron et al. and Goorts et al. found no association between tumor size and pCR [16,17].

Some studies have indicated that ultrasonic characteristics forecast NACT outcomes [17,18]. For instance, Savardis et al. demonstrated that the absence of posterior acoustic shadowing in the US and the lack of spiculation on mammography is associated with higher pCR rates [5]. However, other studies indicate that pCR is unpredictable across all imaging modalities. Different imaging modalities are equally effective at predicting the presence or absence of tumor tissue, which is cCR, according to Croshaw et al., but not at predicting pCR [19]. Other studies reported that just 13% to 25% of cases showed a correlation between a pathologic complete response and a physical exam, mammogram, and breast ultrasound [20-22]. Our study falls under the latter category, where accurate prediction is probably not possible due to the low number of cases.

Radiology studies continue to demonstrate inconsistencies in the imaging features as predictors of pCR. A recent survey of MRI imaging proved that MRI post-NACT remains inaccurate in replacing pCR [23]. On the other hand, studies have shown that tumor grade and hormonal status play a role in pCR rates. pCR occurs more frequently with higher grade tumors, HER-2 negative, and triple-negative cancers [5,15]. Others concurred with a poorer response in hormonal-positive cases [13]. This is probably because triple-negative cancers respond better to NACT than hormonal-positive cases [3].

Unfortunately, we found no such correlations within our cases due to the small sample size in the pathologic sub-group by grade and hormonal status. Different studies additionally concluded that tumor size might be a clinical determinant of pCR. Eventually, the factors that are significantly associated with pCR are hormonal status, grade, lymph node status, and molecular subtyping, which have the strongest correlation in addition to the cancer stage [1,16].

Our study's limited sample size at a single institution and retrospective design are its primary drawbacks. We did not include other imaging modalities. We did not analyze post-treatment changes in US features, because most of our patients did not have follow-up US studies post-NACT. This is because in our institution, US is not routinely performed in the post-NACT assessment and the main objective of the study was to assess the baseline US features and their correlation to the treatment response. A multi-centric study would increase the number of participating patients, and the inclusion of different regions of Saudi Arabia would better reflect breast cancer behavior and pCR in our area. Additional research will need to compare ultrasonic features to other popular imaging features (MRI and mammography) for a more accurate assessment of response to NACT. The presence of post-NACT US would better correlate pCR with cCR and may aid in pre-operative planning. New imaging technologies such as contrast-enhanced US might provide additional insight into tumor behavior with NACT and potential new variables that can predict pCR [24].

## Conclusions

In conclusion, our results showed that among clinical features, younger patient age was the only significant factor associated with pCR in the univariate analysis; however, after multivariate analysis, no statistically significant correlation was observed. Except for tumor size, no other ultrasound features held any statistically significant correlation with pCR. Future studies with multicentric, large sample sizes, and prospective designs are warranted.
